# Constitutional Mismatch Repair Deficiency (CMMRD) Syndrome: A Case Report of a Patient With Multiple Metachronous Malignancies

**DOI:** 10.7759/cureus.41870

**Published:** 2023-07-14

**Authors:** Sameen Bin Naeem, Naqib Ullah, Mussadique Ali Jhatial, Shakeel Muzaffar, Mansoor Abbas, Imran Iftikhar, Ahsan Jameel, Rizwan Masood Sheikh

**Affiliations:** 1 Medical Oncology, Shaukat Khanum Memorial Cancer Hospital and Research Centre, Lahore, PAK; 2 Medical Oncology, King Faisal Specialist Hospital and Research Centre, Riyadh, SAU

**Keywords:** osteochondroma, astrocytoma, non-hodgkin lymphoma, colon cancer, mis match repair deficiency

## Abstract

Defective repair of DNA when heterozygous leads to Lynch syndrome (LS) which is inherited in an autosomal dominant fashion. When homozygous, defective repair of DNA leads to constitutional mismatch repair deficiency syndrome (CMMRD), inherited in an autosomal recessive fashion with a predisposition to develop a pattern of childhood malignancies including hematological and solid cancers. We report such a case of a 21-year-old male who developed anaplastic astrocytoma, Burkitt lymphoma, osteochondroma, and colon cancer successively. Each cancer was treated successfully except for colon cancer which developed liver metastasis after the initial treatment with curative intent. However, the patient has been treated for liver metastasis with curative intent and is currently on follow-up. This case report highlights the importance of maintaining a low threshold for investigating CMMRD and other potential cancer predisposition syndromes when a patient presents with multiple cancers in the early years of their life.

## Introduction

DNA mismatch repair (MMR) mechanism plays a key role in maintaining genomic integrity. The genes MLH1, MSH2, MSH6, and PMS2 are crucial for the correction of base-base and insertion/deletion mismatches generated during DNA replication and recombination, thereby preventing mutations from being permanent in dividing cells [1]. Inactivation in one of these genes can lead to various types of cancer in humans [2,3].

Inherited pathogenic, mono-allelic mutations in one of the four MMR genes can lead to Lynch syndrome (LS; autosomal dominant) with increased risk of colorectal cancers, endometrial cancers, and other malignancies in the fourth and fifth decades of life [3]. LS-associated tumors can also develop due to somatic loss of remaining wild-type alleles, leading to DNA damage and microsatellite instability (MSI) [4].

Some individuals carry bi-allelic deleterious germline mutations in MMR genes, leading to a recessive constitutional mismatch repair deficiency (CMMRD) syndrome. CMMRD is now recognized as a distinct childhood cancer predisposition syndrome. The characteristic tumor spectrum of this syndrome can be divided into four main groups: hematological malignancies, brain tumors, LS-associated tumors, and other malignancies [5]. As CMMRD is still poorly recognized and under-diagnosed, it is rarely suspected at the diagnosis of the first malignancy [6,7]. There are a limited number of cases of CMMRD reported from Pakistan [8,9].

We report the case of a patient with CMMRD syndrome who developed three unusual tumors (anaplastic astrocytoma, Burkitt lymphoma, and colorectal carcinoma) and a benign bone tumor (osteochondroma). Literature was reviewed, and recommendations for surveillance and follow-up are proposed.

## Case presentation

**Figure 1:**
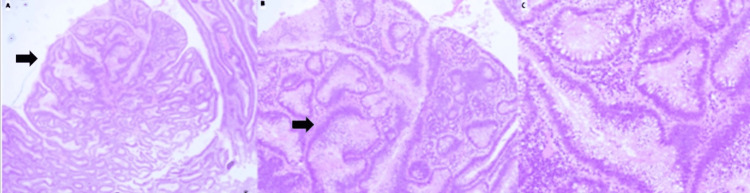
Rectal polyp (H&E) (A) This image of a rectal polyp shows closely packed back-to-back glands in a tubulovillous architecture. (B&C) At higher magnification, the epithelial lining shows low-grade dysplastic changes, with tall columnar cells having pencillate, hyperchromatic nuclei (arrows). No high-grade cytological atypia was noted.

A 21-year-old male, previously treated for anaplastic astrocytoma of the brain 16 years ago (treated with surgery, chemotherapy, and radiation), came from a tertiary care center to our hospital in 2008 under pediatric oncology care with complaints of abdominal pain for three months. He was diagnosed with Burkitt lymphoma, stage 3B, with no evidence of residual or recurrence of anaplastic astrocytoma in the brain. He achieved a complete metabolic response with chemotherapy given as per UKCCSG B-NHL Group B guidelines (cyclophosphamide, vincristine, prednisone, doxorubicin alternating with cytarabine, high-dose methotrexate, and cyclophosphamide). The patient completed five years of active surveillance and was discharged. He presented in 2017 with a left thigh mass which was diagnosed as an osteochondroma. He was referred to an orthopedic surgeon for excision. In 2019, he again presented in the adult oncology clinic with complaints of abdominal pain for one month. There was no evidence of hyper or hypopigmentation on the skin of the trunk or extremities. Further workup revealed multiple descending colon and rectal polypi. Histopathology suggested tubulovillous adenomata with low-grade dysplasia and moderately differentiated adenocarcinoma of the ascending colon (Figure 1).

**Figure 2:**
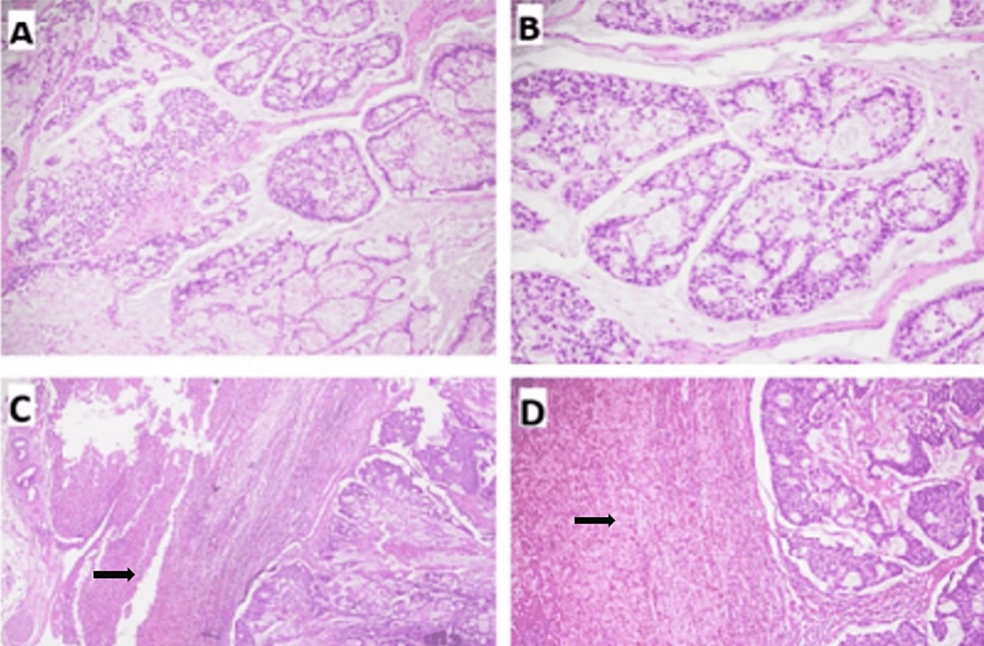
Metastatic adenocarcinoma involving the liver (H&E) (A&B) These photomicrographs show a malignant tumor made up of glands and nests with a cribriform pattern. Tumor cells show moderate cytological atypia and there is extensive extracellular mucin in the background stroma. (B&C) The tumor extensively invades liver tissue (arrows).

He underwent a right hemicolectomy (pT3N1a: AJCC 8th edition). He received chemotherapy (capecitabine/oxaliplatin) for five cycles, followed by completion colectomy with ileorectal anastomosis. Multiple tubulovillous adenomata were identified throughout the colon, however, none of them showed high-grade dysplasia or invasive adenocarcinoma. The patient remained under active surveillance with regular endoscopies and imaging. On follow-up imaging in December 2021, he was found to have hepatic hypo-dense lesions with involvement of adjacent right-sided hepatic ducts. He underwent open total right hepatic lobectomy with cholecystectomy; histopathology was consistent with metastatic adenocarcinoma of the colon (Figure 2).

**Figure 3:**
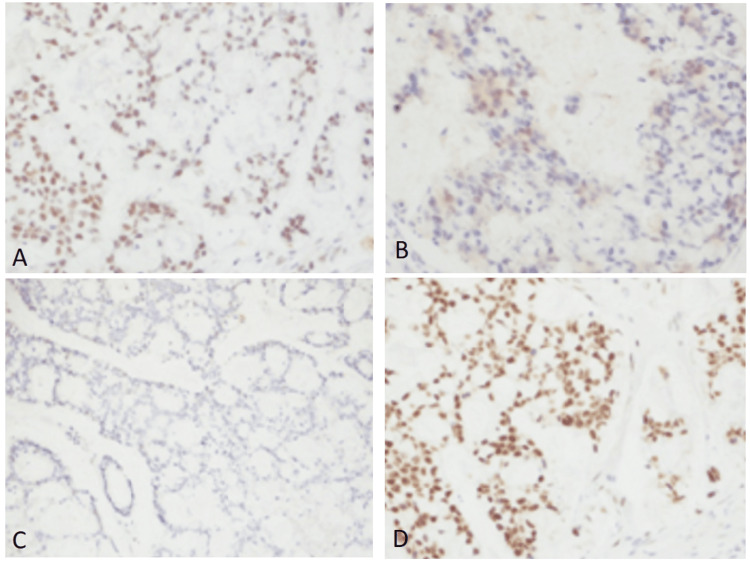
Microsatellite instability testing by immunohistochemistry Immunohistochemical stains for MLH-1 (A), MSH-2 (B), PMS-1 (C), and MSH 6 (D) showed intact nuclear expression. PMS-2 showed weak nuclear expression but it was considered retained as staining was seen in more than 1% of tumor cells.

On the basis of a history of multiple cancers, and the fact that it is rare for adolescents to develop colon cancer and suspicion of hereditary cancer predisposition syndrome, the primary physician decided to rule out MMR deficiency syndrome. The MMR proteins turned out to be stable per immunohistochemistry testing (Figure 3).

However, exome-based next-generation sequencing performed on the histopathology specimen revealed a high tumor mutation burden (74.91 mutations/megabase), MSI-high with a germline, bi-allelic, pathogenic loss of function mutation of MSH6, and a loss of function mutation (p.R2763*(c.8287C>T) in ataxia-telangiectasia mutated (ATM) kinase. In December 2022, on routine follow-up imaging, he was found to have two foci of skeletal metastasis. Upon biopsy, one of the foci turned out to be mucinous adenocarcinoma, consistent with the diagnosis of recurrent metastatic colon cancer. He was considered for immunotherapy, but his family could not afford it. Therefore, he is being considered for palliative chemotherapy.

Follow-up

At the latest follow-up, 17 years after brain tumor removal and 14 years after Burkitt lymphoma, brain MRI and CT imaging suggested no recurrence. As far as colorectal carcinoma is concerned, the patient underwent complete colonic resection in 2019. However, the patient developed liver metastasis in February 2022 for which he underwent a right liver lobectomy. Follow-up imaging suggested a recurrence of colorectal cancer with skeletal metastases which was confirmed on histopathology. He is being considered for palliative chemotherapy.

## Discussion

This syndrome goes by different names, with CMMRD being the most appropriate one. Jacques Turcot first reported two siblings with numerous colorectal adenomatous polyps (familial adenomatous polyposis), colorectal carcinoma, and malignant brain tumors in 1959 [10]. In subsequent years, patients who had multiple colorectal adenomas associated with brain tumors were combined under Turcot syndrome, with underlying germline mutation either in the adenomatous polyposis coli (APC) gene or one or more of the MMR genes [11]. CMMRD is the most suitable term as it also refers to the underlying germline bi-allelic defect in DNA MMR.

Wimmer et al. reported the median age (at diagnosis of CMMRD-related tumors) as follows: brain tumors, 9.5 years; hematological malignancies, five years; LS-associated cancers, 16 years. The mean age at first diagnosis was nine years [5]. Our patient first developed a brain tumor (anaplastic astrocytoma) at the age of 5 years, followed by a hematological malignancy (Burkitt lymphoma) at the age of 8 years, and finally, LS-associated colon cancer at the age of 19 years. He also developed a benign bone tumor at the age of 17 years which is not documented in the literature to be related to CMMRD.

Wimmer et al. also published proposed criteria for testing cancer patients with multiple malignancies for possible CMMRD (Table 1). CMMRD should be suspected at a minimum score of 3 [5].

**Table 1:** Scoring system to determine germline testing eligibility for constitutional mismatch repair deficiency syndrome (CMMRD) [5]

Indications for testing for CMMRD in a cancer patient ≥ 3 points.	
Cancers/pre-cancers: One is mandatory, and add points if more than one is present in the patient.	
Cancer from the Lynch syndrome spectrum of cancers at age < 25 years	3 points
Multiple bowel adenomata at age < 25 years and absence of APC/MUTYH mutation(s) or a single adenoma with high-grade dysplasia at age < 25 years	3 points
WHO grade III or IV glioma at age < 25 years	2 points
Non-Hodgkin lymphoma of T-cell lineage or supratentorial primitive neuroectodermal tumor at age < 18 years	2 points
Any cancer at age < 18 years	1 point
Additional features: optional; if more than one of the following are present, add the points.	
Clinical signs of neurofibromatosis 1 and/or ≥ 2 hyperpigmented and/or hypopigmented skin alterations > 1 cm	2 points
Diagnosis of Lynch syndrome in a first-degree or second-degree relative	2 points
Carcinoma from Lynch syndrome spectrum before the age of 60 years in 1^st^, 2^nd^, or 3rd-degree relative	1 point
A sibling with carcinoma from the Lynch syndrome spectrum, high-grade glioma, supratentorial primitive neuroectodermal tumor or non-Hodgkin lymphoma	2 points
A sibling with any type of childhood cancer	1 point
Multiple pilomatricomas in the patient	2 points
One pilomatricoma in the patient	1 point
Agenesis of the corpus callosum or non-therapy-induced cavernoma in the patient	1 point
Consanguineous parents	1 point
Deficiency/reduced levels of immunoglobin G2/4 and/or immunoglobin A	1 point

Recently, Aronson et al. [12] published diagnostic criteria for CMMRD and recommendations from the international consensus working group proposed six diagnostic criteria for surveillance and familial genetic counseling (Table 2).

**Table 2:** Diagnostic criteria for constitutional mismatch repair deficiency syndrome (CMMRD) [8]

Criterion		Germline result PMS2, MSH6, MSH2, MLH1	Positive Ancillary testing	Clinical Phenotype
Definitive Diagnosis (strong evidence of CMMRD)	1	Bi-allelic pathogenic variants (P/P), confirmed in trans.	One required if patient remains unaffected up to 25 years of age.	Not required if up to 25 years of age (if no malignancy over age 25, ancillary testing required)
	2	Bi-allelic Pathogenic / Likely Pathogenic (P/LP) or LP/LP variants, confirmed in trans.	(a) One required OR (b) Two required if unaffected by a hallmark cancer	(a) Hallmark CMMRD cancer diagnosis OR (b) Two ancillary tests required if meeting the Care for CMMRD criteria of 3 points
	3	Heterozygous P or LP variant (+/- variants of uncertain significance [VUS] or likely benign variants)	One required	Hallmark CMMRD cancer diagnosis
	4	No P or LP MMR variants (including VUS/VUS) or no testing available (i.e., deceased proband)	Two required	Hallmark CMMRD cancer diagnosis
Likely Diagnosis (Moderate evidence of CMMRD)	5	Bi-allelic P/LP or LP/LP variants confirmed in trans	Not required	Care for CMMRD criteria of 3 points
	6	Heterozygous P or LP variant or no testing available (i.e., deceased proband)	Two required	Care for CMMRD criteria of 3 points Individual < age 18 with NF1 features (i.e., no malignancy or polyposis history) Malignancy under the age 30

Clinicians should have a high suspicion of CMMRD if a patient develops a brain tumor at an early age followed by a second hematological malignancy or an LS-associated tumor later in life. Appropriate testing is required to increase the number of patients identified at the time of diagnosis of their first malignancy. Even though this syndrome has a high mortality, screening with early detection of CMMRD-related cancers at an operable stage can lead to a better prognosis. Several authors have emphasized the need to screen the high-risk population [13,14]. Therefore it is important to make early diagnosis for clinical surveillance and survivorship.

Genetic counseling should be offered to parents prior to testing the affected child. Once a homozygous mutation has been identified, heterozygous family members should be followed as per current LS guidelines [15]. Due to the wide range of malignancies in CMMRD patients, key recommendations for monitoring affected patients remains a challenge. Early diagnosis of CMMRD followed by regular cancer screening increases the frequency of detecting related cancers (such as colon and brain tumors) at an operable stage. In theory, this screening includes regular checks such as clinical evaluation, complete blood count and blood test for carcinoembryonic antigen (CEA), brain magnetic resonance imaging, lower gastrointestinal endoscopy and endometrial sampling, and transvaginal ultrasound for endometrial and ovarian cancer in females. However, these recommendations are based on clinical judgment only and do not represent the standard of care.

## Conclusions

Although rare, CMMRD and other cancer predisposition syndromes should be considered and investigated when a patient presents with multiple malignancies during the early years of their life. Surveillance and screening, as appropriate, can help diagnose cancers at an early stage and help minimize morbidity and prolong life.
